# To Our Readers

**Published:** 1896-01

**Authors:** 


### TO OUR READERS.

One of the leading features for 1896 will be a monthly abstract of all the leading features of the German veterinary periodicals. Arrangements have been completed for this work with one of the leading German instructors in this country. In addition our corps of contributors will enable us to give to our readers choice extracts from a number of other foreign journals. Our list of over one hundred contributors to the Journal in 1895 will be doubled in 1896. Other special features of the Journal will be shortly announced, and our constant aim will be to lead veterinary journalism in America.

We beg the indulgence of many of our contributors this month, in that we have been forced to lay over until February an unusually large amount of interesting material with which we have kindly been favored. We have for nearly six months added to each issue from ten to twenty extra pages of reading material and crowded our pages to the utmost they would hold,

ARMY MEDICAL LIBRARY WASHINGTON, D. C. U. S. A.

but so generously have the efforts of the JOURNAL been seconded by its rapidly enlarging corps of contributors that we feel this explanation is again due those whose contributions have not as yet been published.

